# Annotations

**Published:** 1901-10-12

**Authors:** 


### ANNOTATIONS.

### Youth versus Experience.

Whatever men may say about the profession being overcrowded, there seems but little doubt that in one sense there is a scarcity of doctors. In his introductory address at University College Hospital, Dr. Risien Russell said that during the last seven years there had been so great a falling off in the number of men entering the medical profession that there were now 1,660 fewer men qualified to practise medicine than there would have been had the same number joined the profession each year since as did in the year 1893. In the meantime, as is shown by the census returns, the population of the country has increased during the past decade by over 3,500,000. Not only so, but the colonies continue to attract a considerable proportion of those that qualify, and South Africa has drawn away a very considerable number. In addition to all this the action of the General Medical Council, in putting a stop to the employment of unqualified assistants, has had a great effect in providing openings for the junior ranks of the profession. Perhaps Dr Risien Russell is right in the rosy view he takes of the prospects of those who enter the profession at the present time. All the same we hear complaints on every side of the hardness of the times and the difficulty of making ends meet, and we have the impression that in addition to the causes mentioned by Dr. Risien Russell, there are still other factors at work which tend to improve the prospects of young doctors—while they remain young. In every profession, as in every trade, the stress and strain of modern life has created a demand for youthful labour, while the torrent of new ideas, the publicity given to new modes of treatment, together with the ever-growing notion that what is newest is also best, have all led to the undermining of that faith in experience, and that trust in mere seniority which used to be such a stand-by to the elder members of the profession. "Go up thou baldhead" aptly expresses the feeling of the age. "Ripe experience" is no longer a virtue in a physician. What people now seek is a young man "fresh from the schools" and cram full of the most recent "tips." And so it is that youth, more than ever, has its day, and, unless they have succeeded in making a great name, the seniors suffer. This, we fancy, is at least one of the causes of the apparent contradiction that while the supply of doctors turned out by the schools seems hardly equal to the demand, those already within the profession find it difficult sometimes even to make bread and butter.

### The Lack of Sanitary Organisation in London.

The gradual spread of small-pox throughout London will, it is to be hoped, have the effect of drawing attention to a defect in the organisation of the sanitary services of the metropolis which we have again and again pointed out, namely, the lack of any one central authority charged with the duty of initiating and organising the measures necessary for the control of epidemics and provided with the power of enforcing such measures throughout the whole of the metropolitan area. Fortunately there is a good deal of friendly, although entirely unofficial co-operation between the medical officers of the various boroughs, and between them and the health authorities at the central offices of the County Council, and fortunately again Englishmen are apt in emergencies to pay but small respect to the exact letter of the law, so that things do not in all cases work out quite so badly as they would under a *régime* of strict conformity to legal requirements. But we ought not to have to depend upon mere friendly arrangements, nor to be driven to ask our officials to overstep their powers. We have lately heard much about the energy and smart initiative of certain sanitary officials in catching the hop-pickers on their return from the country, inspecting them, and shipping off to hospital those who were found to be suffering from small-pox. No doubt a good bit of work, for doing which the inhabitants of London ought to be very grateful to them. We question, however, whether it was legal, and we have our doubts whether either the railway authorities, the medical officers, or the police had the slightest right to interfere with any of these hop-pickers, or to prevent them and their infected children returning straight to their lodgings in the heart of London. In default of possessing a central controlling authority with power to act London has to trust to the zeal of its officials, and to take its chance that one or other of them will be sufficiently plucky to step outside the strict line of his duty and risk the reprimand with which his enthusiasm may be rewarded. But things ought not to be so, and there certainly ought, in this great city, to be some central authority with full power to act in all emergencies.

### Engaging the Doctor.

When a young woman who finds herself in an "interesting condition" goes to engage the doctor she no doubt thinks it a somewhat disagreeable ordeal; but that is all, and probably the practitioner who is engaged makes even less to do about it. When once the case is "booked" there is an end of the matter until the night-bell rings. But all this is very casual, and, if what we are told about the importance of early diagnosis of morbid conditions is true, surely it is all very wrong. Speaking recently on the subject of the falling birth-rate, Professor Byers said that one way of attacking this serious problem was by taking increased precautions when a woman was pregnant that she should give birth to a living and a healthy child. He thought that we ought to take more care than is usually taken to combat those conditions which are dangerous to a child's life before and during its birth, and he urged that when a pregnant woman, especially one expecting her first child, engages a doctor she should be examined as carefully as if she were an applicant for life insurance. He further urged that towards the end of gestation a most careful abdominal examination should be made, and that if there should be the slightest suspicion of deformity the pelvis should be most carefully measured. In all of this he is evidently in the right. In dealing with eclampsia, or with contracted pelvis, or with cases in which tumours cause obstruction fore-knowledge is everything. It is clear that if a woman is to obtain all the advantage which the attendance of an expert can offer her and if she is to receive all the benefit which can be derived from recent advances in the obstetric art, the ceremony of engaging the doctor must be taken a good deal more seriously than is usually the case.

